# Cluster Headache: Economic and Psychological Burdens of Diagnostic Errors and Delays

**DOI:** 10.7759/cureus.115239

**Published:** 2026-08-26

**Authors:** Vera Gibb, Amber E High

**Affiliations:** 1 School of Nursing, The University of Texas Medical Branch at Galveston, Galveston, USA; 2 Anesthesiology, School of Nursing, The University of Texas Medical Branch at Galveston, Galveston, USA

**Keywords:** cluster headache, diagnostic delay, diagnostic error, headache disorders, headache medicine, patient advocacy, primary headache disorders, suicide headache, suicide risk, trigeminal autonomic cephalalgia

## Abstract

Cluster headache (CH) is a devastating primary headache disorder and is widely recognized as one of the most severe pain conditions encountered in clinical practice. The goal of this narrative review is to examine the challenges contributing to the delayed recognition and diagnosis of CH. We divided the cost of CH into direct and indirect costs. We found that direct costs include healthcare services, such as emergency department, primary care, and specialist visits, diagnostic tests, pharmacy services, oxygen therapy, and neuromodulation devices, and increased healthcare utilization, including more frequent emergency department visits. Indirect costs include such invisible burdens of the disease as pain, demoralization, and suicide risk. Despite the cyclic nature of the disease, CH is a chronically disabling disease that impacts patients even during periods of remission. Misdiagnosis is common, particularly among female patients. Diagnostic delays often impede initiation of optimal treatment. Reducing diagnostic delays will require educating clinicians who may encounter patients suffering from CH, including those in primary care, emergency medicine, pain management, neurology, otolaryngology, and dentistry. To address misdiagnosis, it is recommended to incorporate the Erwin Test for Cluster Headache (ETCH) into clinical practice and healthcare professional education to support earlier recognition and improve diagnostic accuracy. Collaboration among healthcare professionals, patient advocacy organizations, and patients themselves, together with improved diagnostic tools and expanded access to care, may increase awareness, reduce diagnostic delays, and improve patient outcomes.

## Introduction and background

Cluster headache (CH) is a devastating primary headache disorder. It was nicknamed the “suicide headache” due to the severity of pain and suicidal ideation during the attacks. The pathophysiology of CH is not fully understood, and the precise mechanisms remain debated [1]. There is no diagnostic biomarker for CH, and the diagnosis is based on clinical criteria from the International Classification of Headache Disorders [2]. There is a significant diagnostic delay, with a recent report by Van Obberghen et al. [3] estimating an overall diagnostic delay for CH of more than 10 years, resulting in delayed optimal treatment [1]. Patients tend to consult multiple specialties before receiving the correct diagnosis. CH is often diagnosed by neurologists, although one study reported 41% of patients were misdiagnosed by neurologists [2]. The overall rate of misdiagnosis is 49%, with females being misdiagnosed more frequently [4]. Misdiagnoses and diagnostic delays result in increased morbidity, inappropriate treatments, and higher utilization of healthcare services, including emergency department (ED) visits and diagnostic services.** **Given the diagnostic delays and misdiagnoses, a reliable diagnostic tool may be helpful [2] to decrease CH burden, improve patient outcomes, and decrease overutilization of healthcare services. We searched the literature in February-August 2025 using several databases, including CINAHL Complete, PubMed, and Medline, using the search terms cluster headache, diagnostic delay, diagnostic error, primary headache disorders, suicide headache, and trigeminal autonomic cephalalgia. A total of 25 studies were selected. This narrative review of the diagnostic challenges and consequent burdens faced by patients examines the clinical, economic, and psychosocial consequences of delayed CH diagnosis and discusses strategies to improve earlier recognition, including clinician education, diagnostic screening, patient advocacy, and access to specialty care. The review aims to educate healthcare providers about the burden of CH and provide a screening tool to improve diagnostic capabilities. We start by breaking down the direct and indirect costs of CH as well as the invisible burden of CH. Then, we identify patterns and contributors to diagnostic delays and provide tools and resources that may be used to address these barriers.

Direct and indirect cost of CH

Both direct and indirect annual costs of CH in the United States, as well as in other countries, are substantial. The patient impact and the associated societal burdens are enormous.

Indirect costs include lost productivity and income due to absenteeism, short-term and long-term disability, significantly reduced quality of life, impaired social, personal, and professional relationships, and increased suicide risk. Work productivity during cluster cycles is reduced by 65% [4-6]. One study showed that 20% of patients living with CH had lost a job because of their disease, and another 8% of patients were either on disability or were unemployed [7].

Direct costs include healthcare services such as ED, primary care, and specialist visits, diagnostic tests, pharmacy services, oxygen therapy, and neuromodulation devices. Direct costs vary greatly based on the chronicity, frequency, and severity of cluster attacks, as well as access to specialized headache care; the higher the chronicity, frequency, and severity, the greater the costs. Of note, Negro et al. found that patients with chronic CH had 5.4 times higher healthcare costs, more provider visits, diagnostic tests, and drug utilization than patients with episodic CH [5]. Another study found the average number of yearly ED visits was more than four times higher for patients with CH compared with controls (4.5 vs 1.1), and the average number of yearly outpatient visits was more than 3.5 times higher for patients living with a diagnosis of CH compared with controls (25.4 vs 6.9) [8].

While the relationship between headache disorders and increased healthcare utilization is not fully understood, Polson et al. evaluated 4,174 patients over a 3-year period and found that patients with CH incurred substantially higher healthcare costs and utilized healthcare services more frequently than matched controls [9]. Mean medical costs were 155% higher ($25,805 vs. $10,140), pharmacy costs were more than double ($9,197 vs. $4,368), and patients were 2.3 times more likely to fill an opioid prescription. The study also found significantly higher healthcare utilization across all service types, with higher costs for emergency department, hospital outpatient, physician office, and home infusion/specialty medication services. Overall, patients with CH utilized healthcare services at a significantly higher rate and incurred substantially greater healthcare costs than matched controls. These findings highlight the considerable healthcare burden associated with CH and underscore the importance of timely diagnosis and appropriate treatment.

There is an unmet need to improve outcomes and contain costs for patients with CH [9]. Ford et al. found that 23% of patients with CH were hospitalized [10]. Overall, CH impacts all aspects of a patient’s life, including multimorbidity of somatic and psychiatric diseases [4]. Of the 55 health conditions examined by Joshi et al., smoking, depression, dental disorders, and deviated septum were significantly more common in patients with CH compared with controls [8]. The estimated total direct cost of CH in the U.S. is greater than $2.8 billion/year [11].

Invisible cost of CH: pain, demoralization, and suicide risk

Despite the cyclic nature of the disease, CH is a chronically disabling disease psychologically and physiologically that impacts patients even during periods of remission [4]. In a large international survey, respondents rated CH pain as more severe than several other intensely painful conditions, including childbirth, gunshot wounds, and nephrolithiasis [12]. There is a higher prevalence of anxiety and depression in patients with CH compared to controls [4], and the recurrent painful, intolerable attacks are associated with depression, disability, and desperation. The debilitating condition has been linked to increased risk of suicide since its initial descriptions by the American neurologist, B.T. Horton, in 1939. Of note, the study setting, patient selection, definitions, and assessment methods influence the statistics, resulting in substantial variability of estimates of suicidal ideation across studies (e.g., lifetime versus point/period prevalence, tertiary headache-center populations versus broader populations, and differing definitions or instruments). Compared with the general population, patients with CH experience a greater mental health burden and higher rates of suicidality. Patients with CH have a significantly higher prevalence of lifetime active suicidal ideation (47.0% vs. 26.7%), high suicide risk (38.0% vs. 18.5%), lifetime history of depression (67.0% vs. 32.6%), and demoralization (28.0% vs. 15.6%) [13]. Suicidal ideation in patients with CH was not associated with depression but with demoralization, a state of profound hopelessness, feelings of being trapped, and inability to cope. While demoralization and depression often co-occur, they are two distinct entities. In depression, while motivation is lacking, the appropriate course of action is known; with demoralization, there is no appropriate course of action, and the absence of hope is the central theme [13]. Patients with chronic CH are more likely to be demoralized than patients with episodic CH [13]. Stigma compounds the burden even more [14]. Another study showed the overall rate of suicidal ideation in patients with CH at 8.0% and the overall rate of suicide attempts at 1.2% [15]. However, patients seen in tertiary headache centers show a much higher rate of suicidal ideation and rate of suicide attempts estimated at 44.6% and 5.1%, respectively [15]. In patients with CH, the risk of demoralization and suicide is also increased due to the high rates of misdiagnosis [13]. The evidence of a robust and persistent association of headache diagnoses with suicidal ideation and attempts [16] raises a question of accurate diagnosis.

This article was previously presented as a poster at the American Headache Society’s 67th Annual Scientific Meeting on June 19, 2025, in Minneapolis, MN.

## Review

Diagnostic delays in CH 

CH can begin during childhood or adolescence, and younger age at onset has been associated with greater diagnostic delay [17]. The clinical presentation of CH in children, adults, and the elderly is similar [18]. Currently, no imaging or molecular diagnostic biomarkers are available [2], and the diagnosis is made using clinical criteria from the International Classification of Headache Disorders [19].

Diagnostic criteria for CH

Per the International Classification of Headache Disorders, 3rd edition [19], Section 3.1, diagnostic criteria for CH include at least five attacks fulfilling criteria B-D. Attacks are characterized by severe or very severe unilateral orbital, supraorbital, and/or temporal pain lasting 15-180 minutes when untreated. The headache is accompanied by at least one ipsilateral cranial autonomic symptom, including conjunctival injection or lacrimation, nasal congestion or rhinorrhea, eyelid edema, forehead or facial sweating, or miosis or ptosis, and/or a sense of restlessness or agitation. Attacks occur with a frequency ranging from one every other day to eight per day and are not better accounted for by another diagnosis. Additional clinical characteristics of CH are summarized in Table 1.

**Table 1:** Clinical characteristics and common triggers of cluster headache (CH) Source: [20,21]

Characteristic	Description
Clinical presentation	Unilateral, sharp, stabbing pain rapidly reaching an excruciating intensity and typically felt around the orbit and temporal area; pain is described as feeling like a “red hot poker in the eye”
Duration of attacks	Between 15 and 180 minutes
Associated symptoms	At least one of the cranial autonomic symptoms: redness or tearing of the eye, nasal congestion or runny nose, eyelid swelling or drooping, forehead and facial sweating, and/or constricted pupils
Behavioral manifestations during the attacks	Restlessness and agitation
Frequency of attacks	Once every other day, up to eight episodes per day
Cluster cycles	Episodic CH bouts last from a few weeks to a few months
Periodicity	Circadian, with a preponderance of attacks during the sleep phase and circannual, with bouts peaking at seasonal changes, particularly spring and fall
Cluster headache triggers	Medications such as nitroglycerin (vasodilators); alcohol use; tobacco exposure; histamine release; foods that contain nitrates; petroleum; nail varnish

Despite this peculiar phenotype, only a small proportion of patients with CH are correctly diagnosed in a timely manner. The condition often begins during childhood and adolescence but is not diagnosed until adulthood [17]. One study shows that the diagnostic delay was 1-8 years across 13 countries [2].

Predictors of misdiagnoses and diagnostic delay

According to Van Obberghen et al. [3], qualitative analyses also identified several predictors of this diagnostic delay. The presence of cranial autonomic symptoms reduces diagnostic delay, whereas the following factors increase it: younger age at CH onset, alternating attack sides, and nocturnal headaches. Delayed diagnosis is a widespread problem. Even though the time to diagnosis varies from country to country, diagnostic delays are common even in countries with well-developed healthcare services, including the U.S., Japan, and many European countries [22]. Unfortunately, many healthcare providers do not treat CH as a potential emergency with the stroke or seizure level acuity [14].

Before they receive the correct diagnosis, patients consult between two and five clinicians [17], including specialists in neurology, neurosurgery, ophthalmology, otolaryngology, and psychiatry [2]. Patients often receive multiple diagnoses prior to being correctly diagnosed [22]. Although neurologists are often the clinicians who ultimately diagnose CH, one study found that 41% of patients were initially misdiagnosed by neurologists [23]. The most frequent incorrect diagnoses included migraine, sinusitis, trigeminal neuralgia, jaw disease, and dental disorders [22]. Therapeutic mismanagement during the diagnostic delay is common among patients with CH [24]. Misdiagnoses and diagnostic delays result in increased morbidity and contribute to the high costs of ED visits and radiology services [11]. On a positive note, Van Obberghen et al. report a reduction in CH diagnostic delay over time since the 1960s, a trend that continues every decade since 2000 [3].

Improving diagnosis

The Erwin Test for Cluster Headache (ETCH) is a three-item diagnostic screening tool developed to improve the recognition and diagnostic accuracy of CH [2]. Published in 2021, the decision-tree algorithm identified three questions that demonstrated 85% sensitivity and 89% specificity for CH among 224 participants with headache disorders [2]. The three screening questions that comprise the ETCH are summarized in Table 2.

**Table 2:** The three-item Erwin Test for Cluster Headache (ETCH) An affirmative response to all three questions demonstrates 85% sensitivity and 89% specificity for cluster headache. Reproduced from Erwin et al. [2]. Copyright © 2020 The University of Texas Health Science Center at Houston. Reproduced under the Creative Commons Attribution-NonCommercial 4.0 International License.

Question	Response Options
Is this the worst pain you have ever experienced?	Yes / No
Imagine setting a timer. Does the headache last less than 4 hours?	Yes / No
During a headache, do one or more of these happen to you: your eye turns red on only one side; your eye waters on only one side; your nose runs on only one side; or your nose gets congested on only one side?	Yes / No

The first question focuses on the higher intensity of CH pain compared with other intensely painful conditions, including kidney stones, labor pain, and pancreatitis [12]. The second question focuses on pain duration of less than 4 hours compared with other headache disorders, which may last longer than four hours. The third question focuses on cranial autonomic features that are prominent in the clinical presentation of CH [2]. Of note, 15% of CH patients said they had self-diagnosed using different sources of information before seeking medical confirmation [3].

Although the ETCH requires broader external/prospective validation before widespread implementation, the high specificity of this screening tool has promise for the better identification of CH in the general population. The screening tool is easy to use and can be implemented in any clinical setting, including primary care and specialty practices (neurology, pain management, otolaryngology, and dentistry) [2], as well as in medical schools and advanced practice healthcare professional education programs. A positive screening result should prompt appropriate clinical assessment. 

Advocacy, support, and access to care

Although improving provider recognition and expanding use of diagnostic tools, such as the Erwin Test, are important steps toward reducing diagnostic delays, meaningful progress will require collaboration beyond the clinical setting. Patient advocacy organizations have become essential partners in CH recognition, education, and research. Patient advocacy organizations have helped increase public and provider awareness, support the development of diagnostic resources, advocate for improved access to evidence-based treatments such as home oxygen, and foster patient-centered research initiatives [25]. These efforts complement the work of healthcare professionals and have helped drive important advances in the field.

Beyond advancing research and policy, patient advocacy organizations, such as Clusterbusters and the Will Erwin Headache Research Foundation, provide something equally important: a sense of community [25]. These organizations connect individuals living with CH through peer support groups, educational resources, patient conferences, and online communities where patients and caregivers can share experiences and practical guidance [25]. For many individuals, these resources may reduce isolation and provide important psychosocial support in the context of the elevated suicide risk associated with CH, as well as identify experienced headache specialists, navigate evidence-based treatments, and stay informed about current research [25]. Patients who recognize their symptoms through advocacy organizations or peer communities may be better equipped to seek appropriate evaluation, advocate for themselves, and potentially reduce delays in diagnosis and treatment [25]. Advocacy transforms the experience of living with a devastating pain disorder into an opportunity to educate others, improve care, and drive continued advances in diagnosis, treatment, and patient outcomes.

## Conclusions

Cluster headache (CH) is a primary headache disorder notable for its severity of pain and suicidal ideation during attacks. Diagnostic delays and clinical mismanagement of the condition are common. Provider education may improve diagnostic accuracy among providers who may encounter patients with CH, including those in primary care, emergency medicine, neurology, pain management, otolaryngology, and dentistry. Applying the proven screening tools discussed earlier in clinical practice and medical and advanced practice provider education programs may facilitate earlier recognition and a more efficient CH diagnostic process. Ultimately, meaningful reductions in diagnostic delay will depend on a collaborative effort among healthcare professionals, researchers, patient advocacy organizations, and patients themselves to improve awareness, expand access to care, and promote timely diagnosis and treatment. Earlier and more accurate diagnosis is expected to reduce demoralization among patients with CH, provide access to more effective treatments, lessen the impact of this recurrent and severely painful condition, and decrease unnecessary utilization of healthcare services.
